# Factors Affecting Poultry Producers’ Attitudes towards Biosecurity

**DOI:** 10.3390/ani14111603

**Published:** 2024-05-29

**Authors:** Arthi Amalraj, Hilde Van Meirhaeghe, Anne-Christine Lefort, Nathalie Rousset, Justine Grillet, Annick Spaans, Aitor Devesa, Sandra Sevilla-Navarro, Giuditta Tilli, Alessandra Piccirillo, Artur Żbikowski, László Kovács, Mária Kovács-Weber, Ilias Chantziaras, Jeroen Dewulf

**Affiliations:** 1 Faculty of Veterinary Medicine, Ghent University, Salisburylaan 133, 9820 Merelbeke, Belgium; ilias.chantziaras@ugent.be (I.C.); jeroen.dewulf@ugent.be (J.D.); 2 Vetworks BV, Knokstraat 36, 9880 Aalter, Belgium; hilde.vanmeirhaeghe@vetworks.eu (H.V.M.); giuditta.tilli@vetworks.eu (G.T.); 3 ITAVI, Institut Technique de l’Aviculture, Pisciculture et Cuniculture, 75009 Paris, France; anne-christine.lefort@elancoah.com (A.-C.L.); rousset@itavi.asso.fr (N.R.); grillet@itavi.asso.fr (J.G.); 4 ZLTO, 5223 DE s’-Hertogenbosch, The Netherlands; annick.spaans@zlto.nl; 5 Poultry Health Centre of Catalonia and Aragon (CESAC), 43206 Reus, Spain; 6 Centro de Calidad Avícola y Alimentación Animal de la Comunidad Valenciana (CECAV), 12539 Castellón, Spain; s.sevilla@cecav.org; 7 Department of Comparative Biomedicine and Food Science, University of Padua, Viale dell’Università 16, 35020 Legnaro, Italy; alessandra.piccirillo@unipd.it; 8 Department of Pathology and Veterinary Diagnostics, Institute of Veterinary Medicine, Warsaw University of Life Sciences, 02-776 Warsaw, Poland; artur_zbikowski@sggw.edu.pl; 9 Department of Animal Hygiene, Herd Health and Mobile Clinic, University of Veterinary Medicine, H-1078 Budapest, Hungary; kovacs.laszlo@univet.hu; 10 Poultry-Care Kft., H-5052 Újszász, Hungary; 11 Department of Animal Husbandry Technology and Welfare, Institute of Animal Sciences, Hungarian University of Agriculture and Life Sciences, H-2100 Gödöllő, Hungary; kovacs-weber.maria@uni-mate.hu

**Keywords:** ADKAR^®^ model, biosecurity, education, Europe, poultry

## Abstract

**Simple Summary:**

Simple Summary: The prevention of the spread of poultry diseases within and among farms largely depends on the adoption of good biosecurity practices. Through the process of profiling European poultry producers, we were able to identify the particular aspects that influence the adoption of biosecurity practices. The results suggest a wide and varied attitude towards biosecurity in the poultry farming community. Generally, most producers appear to be aware of the risks associated with poor biosecurity and the outcomes of carrying out or not carrying out certain practices. Yet, compared with producers of laying hens or parent breeding stock, meat poultry producers (broiler, turkeys, and ducks) appear to have more blocking components with regard to biosecurity. The education level had a positive effect on perception about biosecurity. Poultry producers are not a homogeneous group, and to change their attitudes and actions, approaches must consider the producers’ mindset and need to be tailored to the specific needs of the producers.

**Abstract:**

Poultry producers’ attitudes towards biosecurity practices were assessed by using the ADKAR^®^ (Awareness, Desire, Knowledge, Ability, and Reinforcement) behavioral change model. Conventional poultry producers (*n* = 155) from different production types including broilers (*n* = 35), layers (*n* = 22), breeders (*n* = 24), turkeys (*n* = 19), ducks (*n* = 23), free-range broilers (*n* = 11), free-range layers (*n* = 11), and hatcheries (*n* = 10) from seven European countries were scored for each ADKAR element (1 = total absence to 5 = perfect fulfilment). Each country performed selected interventions (e.g., coaching, participatory meetings, etc.) to improve biosecurity compliance. After the interventions, significant change was observed in three of the four attitude elements. The overall mean scores ($\bar{x}$ ± SD) obtained during the initial assessment (*n* = 130) were 4.2 ± 0.6 for Awareness, 4.1 ± 0.7 for Desire, 3.8 ± 0.8 for Knowledge, and 4.0 ± 0.7 for Ability, whereas after intervention, the scores were A = 4.3 ± 0.6, D = 4.2 ± 0.7, K = 4.1 ± 0.7, and Ab = 4.1 ± 0.7. The Reinforcement component was only evaluated after the change and obtained a score of 3.7 ± 0.7 on average. Identifying the elements influencing poultry producers and their behavior related to farm management decisions was useful in guiding our educational interventions to effectively change their behavior.

## 1. Introduction

The success of any type of animal production depends, among other things, on the implementation of biosecurity measures. Therefore, understanding how farmers feel about disease prevention is important because it may reveal how inclined they are to change their practices in response to advice [1]. This knowledge could be used to focus activities aimed at encouraging the adoption of biosecurity measures. Little is known about how people make decisions related to biosecurity, why they choose to adopt protective behaviors, and how they stick to those behaviors [2]. Explicit factors such as laws/regulations, cost or financial aid, time, discomfort, and structural barriers do exist [3]. Therefore, internal factors (intrinsic to the individual) like socio-demographic characteristics, such as experience and level of education, age, gender, knowledge, and an understanding of biosecurity principles, also exist [4]. Moreover, farmers’ choices to implement preventive measures are influenced by a variety of other factors, including their personal opinions about the practices and whether they receive tailored guidance from veterinarians [5,6]. 

Despite the fact that farmers are more inclined to adopt biosecurity recommendations given by their veterinarian [7,8], the success of any advice depends on whether it is implemented [5]. According to the Theory of Planned Behaviour (TPB) [9], attitudes and beliefs about an action are linked to the intention to perform and to actually perform that action. For the rigorous and sustainable application of biosecurity measures, a more or less profound change in daily practices is needed. Nevertheless, “change”, for a person, can be a long process full of barriers. The process of the adoption of new practices over time and how they transform into “habits” is not immediately evident [10,11]. Brennan and Christley [7] stated that cattle farmers viewed biosecurity to be more time- and money-efficient than actually treating sick animals. According to some authors [12,13,14], veterinarians considered that on-farm biosecurity measures were not being implemented properly due to a lack of veterinary time, or interest in or knowledge of farm-level biosecurity measures. 

Many determinants influence the implementation of biosecurity measures and compliance with them over time. Better biosecurity compliance can also be linked with specific personality traits. Examples include a sense of self-efficacy (i.e., having confidence in one’s abilities, or feeling capable of performing something), being meticulous, expressing a willingness to succeed [3,4], perception of the problem, and lastly, motivation [15,16,17]. Attitudes play a big role in whether or not farmers make efforts to stop indirect disease transmission channels [1,18].

In this study, we expanded the use of the ADKAR^®^ change management model [19] used in corporate business into animal husbandry practice. ADKAR^®^ is an acronym for Awareness, Desire, Knowledge, Ability, and Reinforcement, identifying the five elements of behavioral change. Poultry producers’ awareness of why the change is needed sets the stage for any further communication that involves reasons to change, scope of the change, and who receives the benefits [20]. Desire or willingness to change is influenced by several factors, for example, motive to keep healthy animals, not being pleased with current farming conditions, or even previous experiences with change [20]. Several authors have stated that a lack of knowledge [4,21], failure to comprehend biosecurity concepts [22,23,24], or a lack of understanding about the risks associated with poor hygiene measures [14] was the primary reason for non-compliance. The ability to adopt new practices focuses on the application of newly learned skills, as well as overcoming potential implementation challenges [20]. During this phase, the poultry producer may need additional expertise to help eliminate obstacles. One of the many barriers to introducing new or alternative management approaches, according farmers, was a lack of time to employ them [25], the expense or non-availability of skilled people, or a lack of proper infrastructure. The last element, “Reinforcement”, represents the sustainability of the established change and was evaluated after the interventions took place.

This model was previously adapted to profile farmers about antimicrobial stewardship in poultry and pig production [26]. Farmer profiling models are practical tools to help herd veterinarians or other animal health advisors to assess perception about biosecurity. Follow-up recommendations will support industry representatives, researchers, veterinarians, and stakeholders and will support policy-makers when motivating farmers to adopt best management practices. 

There is a notion that attitudes affect behavior, giving rise to the belief that we can change behavior if we can first understand attitudes before attempting to change them. In our opinion, barriers to the implementation of biosecurity are insufficiently investigated in poultry farming, and these challenges need to be resolved at the farm level. Therefore, the goals of this study were to investigate the attitudes of poultry producers towards biosecurity and to assess to what extent the implementation of supporting measures may influence these attitudes. 

## 2. Materials and Methods

### 2.1. Adapting the ADKAR^®^ Change Management Model with Regard to Biosecurity Compliance

With an expert’s input, the authors transformed the five-point scale [19] of the adapted ADKAR^®^ model [26] to be utilized in evaluating poultry producers’ attitudes toward change in biosecurity practices. The lowest score on this scale was 1, and the highest score was 5. The individual scores for each element established a poultry producer’s ADKAR^®^ profile. Every element that receives a score of three or lower is considered an element that blocks change [19].

### 2.2. Study Design and Farm Recruitment

The study’s target population were European poultry producers. A longitudinal study was set up between January 2022 and June 2023 on 155 poultry production units, including broiler producers (*n* = 35), layer producers (*n* = 22), breeders (*n* = 24), turkey producers (*n* = 19), duck producers (*n* = 23), free-range broiler producers (*n* = 11), free-range layer producers (*n* = 11), and hatcheries (*n* = 10), in seven countries included in the NetPoulSafe consortium (G.A. 101000728), namely, Belgium, the Netherlands, Spain, Italy, France, Poland, and Hungary. Inclusion criteria were that participants had to be producers of conventional poultry, like enclosed broiler producers, enclosed layer producers, breeding companies, duck producers, turkey producers, free-range broiler producers, free-range layer producers, or hatcheries, and were willing to take part in a six-month biosecurity intervention study. Poultry producers were invited to participate voluntarily in the project via communication targeting poultry farmers, farm managers, advisors, and veterinarians through local agricultural press, newsletters, social media channels (Facebook, LinkedIn, Twitter, and WhatsApp), direct contact (phone or email), and professional connections of the authors.

### 2.3. ADKAR^®^ Profiling of Poultry Producers

ADKAR^®^ profiles were determined for the 155 participating poultry producers. Questions related to the participants’ gender, age, educational background, and satisfaction with work–life balance were also included. Provisions in the questionnaire made it possible to maintain anonymity. Prior to filling in the form, each participant gave their written consent with regards to data management, data storage, and participation to a follow-up intervention. The participants were visited by one and the same facilitator per country, who received prior training to become acquainted with the ADKAR approach and to address any concerns. The facilitator rated each participant on the first four elements (A-D-K-A) in accordance with the guidelines in Table 1 during a farm visit. This was accomplished by questioning the participant’s viewpoint on recurring issues and hygiene management following the completion of a biosecurity audit with Biocheck.UGent^TM^ surveys (https://biocheckgent.com/en/surveys) accessed on 1 January 2022.

### 2.4. Intervention Methods to Effectively Induce Behavioral Change

Following the initial assessment, specific supporting measures were implemented in each partner country for all production types, except hatcheries (Table 2).

In Belgium, Spain, France, Italy, and Hungary, a trained coach offered on-farm coaching sessions using non-directive questioning and interaction [27]. The main goal of this coaching was to develop a feasible improvement plan specific for the farm in agreement with the farmer. To facilitate the achievement of this goal, relevant stakeholders (e.g., farm veterinarian, integrated company representative, external expert, etc.) were invited to participate in the coaching. The interaction among the farmer, the stakeholders, and the coach aimed at the co-creation and co-ownership of the plan to increase the likelihood of implementation of the proposed changes. At the one-day biosecurity workshop in Poland, expert presentations were followed by open debate between the farmers and the experts on the implementation of biosecurity measures and the related challenges and solutions. During participatory workshops [28] in France, farmers and a veterinarian identified one or more common challenges and used thematic maps to discuss solutions to enhance the biosecurity plan. Examples include structural thematic maps (zone defining, fence installation, etc.) and functional thematic maps (incoming and outgoing flow management, hygiene and cleaning and disinfection protocols, flock management, etc.). At the virtual farm tour followed by group discussion in Italy, several farmers from different integrated companies were shown videos of biosecurity practices on other farms, allowing them to visualize various scenarios on their farms and to discuss effective and ineffective biosecurity practices, standard and unfamiliar procedures, challenges, and more. The online sector meetings in the Netherlands addressed the fundamentals of biosecurity and coaching, along with a demonstration of Microsoft Paint 3D and Google Maps for farm-zoning visualization. Subsequently, online individual coaching sessions were provided. In Hungary, a general biosecurity training module was developed and delivered by an expert followed by open debate with the farmers. Subsequent individual coaching sessions were held at the farm. After a duration of six months, the participants’ attitudes were profiled again to identify changes in perception of biosecurity.

### 2.5. Decision-Making Rule for Providing Intervention

Acceptance of any change is believed to be blocked by scores of 3 or less on any of the ADKAR components. In any of the individual coaching sessions, the results of the ADKAR profiling were included in the approach. Whenever the participant scored low (≤3) for the element “Awareness”, the consequences arising from poor biosecurity were discussed. A “why” question for the existing issues on the farm and “why” biosecurity is essential were discussed in detail. For the element “Desire” (≤3), to induce an interest, the benefits of the change were explained. For the element “Knowledge”, depending on a specific problem on the farm, an educative approach was used in the form of visual aids and PowerPoint presentations. For the element “Ability”, low scores were dealt with by discussing topics such as making structural changes and investments towards better biosecurity.

### 2.6. Data Handling and Statistical Analysis

To enable data cleaning and analysis, data were exported into a Microsoft Office Excel document. Statistical analysis was carried out by using IBM^®^ SPSS^®^ Statistics for Windows Version 29 (IBM Corp., Armonk, NY, USA). All responses were analyzed with descriptive statistics. A non-parametric Kruskal–Wallis test, including Bonferroni correction for multiple tests, was used for checking associations between Awareness, Desire, Knowledge, and Ability scores and demographic characteristics (age, gender, and education) and for the comparison of the scores among the different poultry production systems. The Wilcoxon signed-rank paired test was used to evaluate the difference between pre- and post-intervention assessment scores. A *p*-value < 0.05 was used as the level of significance for all tests.

Data from 25 farms were not included in the pre- and post-intervention analyses, since some farms voluntarily withdrew from the study (*n* = 15), and for hatcheries (*n* = 10) no intervention was applied.

## 3. Results

### 3.1. Adapted ADKAR^®^ Profiling Model with Regard to Biosecurity Compliance

The description of the ADKAR^®^ elements and their corresponding score criteria determined by the research team are detailed in Table 1.

### 3.2. Study Sample and Population Characteristics

#### 3.2.1. Descriptive Statistics

Out of the 155 poultry producers, demographic information was provided by 151, 136, and 123, respectively, for the variables age, gender, and education level. According to the study results, the majority of poultry producers (47.7%, *n* = 72/151) were middle-aged (within the age group of 35–50 years), 43 producers (28.5%) were older than 50 years, and 36 producers (23.8%) were younger than 35 years. Over three-quarters of the poultry producers (77.9%, *n* = 106/136) were male, and there were 30 (22.1%) female poultry producers. From the 123 valid responses, 119 poultry producers had completed formal education: 63 (51.2%) with university degrees and 56 (45.5%) with higher education (middle/higher secondary). Four (3.25%) producers had completed up to lower education (primary or minimal secondary school). Of 148 responses, 131 poultry producers (88.5%) reported to be satisfied with their work–life balance.

#### 3.2.2. Association between Age, Gender, and Education and Scores Reflecting Attitudes

The general descriptive information on the scores obtained by the participants and gender, age, and education information are provided in Table 3. The study found that attitude scores were influenced by both the education level and the age group. Significant differences in Awareness levels were observed across the age categories of under 35 and over 50, with a higher percentage of younger producers (97.2%) scoring ≥ 4 compared with older producers (86.0%). Likewise, more younger producers (91.7%) scored ≥ 4 in the element Ability compared with older producers (67.4%; *p* = 0.017). More middle-aged producers (86.1%) scored ≥ 4 in Knowledge, significantly differing (*p* = 0.021) from older producers (67.4%) who scored ≥ 4 in Knowledge. The scores gained for the element Desire did not differ significantly across different age groups; yet, we found more young producers (94.4%) scoring ≥ 4 in Desire when compared with middle-aged (79.2%) and older producers (83.7%). The attitude scores did not differ between the two genders.

The attitude scores were associated with the poultry producers’ level of education. That is, the higher the level of education, the higher the ADKA scores (Table 3). Across the different education groups, a higher percentage of university graduates scored ≥ 4 for all four elements. The Awareness scores were significantly higher (*p* < 0.01) in producers who had completed university (mean ± SD = 4.5 ± 0.6), compared with the lower (mean ± SD = 3.3 ± 0.5) and higher (mean ± SD = 4.1 ± 0.5) education categories. Scores for Desire (mean ± SD = 2.8 ± 1.0) were low in producers in the lower education category, significantly differing (*p* = 0.004) from university education (mean ± SD = 4.3 ± 0.7). A significant difference (*p* < 0.001) was also seen when comparing higher education (mean ± SD = 3.9 ± 0.7) with university education (mean ± SD = 4.3 ± 0.7). Likewise, Knowledge across groups differed significantly (*p* < 0.01) between lower (mean ± SD = 2.3 ± 0.5) and university (mean ± SD = 4.1 ± 0.8) and between higher (mean ± SD = 3.8 ± 0.6) and university (mean ± SD = 4.1 ± 0.8). Ability across groups differed significantly (*p* < 0.05) between lower (mean ± SD = 3.3 ± 0.5) and university (mean ± SD = 4.2 ± 0.7) and between higher (mean ± SD = 3.9 ± 0.7) and university education (mean ± SD = 4.2 ± 0.7).

### 3.3. ADKAR^®^ Profiles of Poultry Producers

The mean scores ($\bar{x}$ ± SD) for Awareness (A), Desire (D), Knowledge (K), and Ability (A) concerning biosecurity obtained by poultry producers of different production types are presented in Table 4. Out of 155 poultry producers, 98 (63%) scored 4 or 5 in each of the first four elements. This included 11/19 turkey producers, 19/35 broiler producers, 14/22 layer producers, 19/24 breeders, 12/23 duck producers, 6/11 free-range broiler producers, 8/11 free-range layer producers, and 9/10 hatcheries. Among them, there were 11 Belgian (61%, 11/18), 14 Spanish (61%, 14/23), 22 Hungarian (73%, 22/30), 15 Polish (71%, 15/21), 18 Italian (69%, 18/26), 9 French (43%, 9/21), and 9 Dutch producers (56%, 9/16). Nineteen of them (12%, 19/155) scored 5 in all four elements, which included producers from five broiler farms (14%, 5/35), two layer farms (9%, 2/22), five breeder farms (21%, 5/24), one free-range broiler farm (9%, 1/11), one free-range layer farm (9%, 1/11), and five hatcheries (50%, 5/10). None of the duck or turkey producers scored 5 in all four elements. Overall, 37% (57/155) of the poultry producers scored 3 or less in at least one of the first four ADKAR^®^ elements. About three percent (4/155) of the poultry producers scored 3 or less in all first four ADKAR^®^ elements. Among them, two were turkey and two were enclosed broiler producers. One out of ten hatcheries had a single blocking element, with an Awareness score of 3. For Awareness, 10.3% (16/155) and, for Desire, 15.4% (24/155) of the poultry producers scored 3 or less. Ten poultry producers scored 3 or less in both Awareness and Desire, reflecting the perception and motivation parts of biosecurity compliance. Meanwhile, low (≤3) Knowledge and Ability scores were obtained in 21.2% (33/155) and 20.6% (32/155) of the poultry producers, respectively. None of the poultry producers with a hatchery (*n* = 10) received a score of 3 or lower for the element Knowledge.

### 3.4. Comparing ADKAR^®^ Profiles among Different Poultry Production Types

The ADKAR profiles of the farmers differed across the poultry production types (Table 4). Producers from breeding companies had a significantly (*p* = 0.021) higher biosecurity Awareness score compared with turkey producers. Producers from hatcheries scored higher in Desire than duck producers (*p* = 0.037). The scores for Knowledge were significantly higher among hatchery producers compared with producers of duck (*p* = 0.032) and turkey (*p* = 0.035). Lastly, for the element Ability, hatchery producers received significantly higher scores compared with duck (*p* = 0.006) and turkey (*p* = 0.044) producers. Likewise, Ability scores of breeders was significantly (*p* = 0.024) higher than those of turkey producers.

### 3.5. ADKAR^®^ Profiles of Poultry Producers before and after Intervention

Table 5 presents the scores ($\bar{x}$ ± SD) received by 130 poultry producers before and after the application of supporting measures. The scores for Awareness, Desire, and Knowledge significantly improved after the intervention. For the different poultry production types, scores either improved or remained unchanged.

## 4. Discussion

Several studies focus on farm-level biosecurity practices in poultry production concerning specific diseases [29,30,31,32,33,34,35] and address the issue of what should be performed [36] rather than why biosecurity is not practiced. This study investigates the attitudes concerning the five key elements in designing effective change with regard to biosecurity. For this purpose, we adapted the ADKAR^®^ change management model as an assessment and supportive tool when implementing intervention strategies to enhance biosecurity procedures. This is the first time an extensive examination was performed to understand the attitudes and behaviors of different kinds of poultry producers spread over seven countries in the European Union. Having multiple countries represented in the study is an important asset, as it captures possible regional diversities. However, it is important to emphasize that the farms were not randomly selected in each country and that the number of farms per country and production type was limited. Therefore, this study does not allow for making country-specific conclusions.

By integrating basic biosecurity measures into daily farm operations and making them standard procedures [37], farmers can significantly reduce the risk of disease outbreaks [36,38] and maintain a healthy and productive farming environment [39,40,41]. Nonetheless, multiple studies have shown that poultry producers do not engage in numerous biosecurity practices [34,42,43,44,45,46,47], and several explanations have been put forth.

The results of this study show that according to the ADKAR^®^ change management model, 57 out of the 155 poultry producers had at least one barrier to change, whereas no effective barriers were seen in the other 98 producers. For instance, all but one of the hatcheries had ADKA scores that were higher than 3, suggesting that the managers of hatcheries already had good levels of Awareness, Desire, Knowledge, and Ability to implement the necessary changes related to biosecurity practices and required no extra effort to be addressed. More blocking elements were identified in producers who raised meat poultry (broilers, turkeys, and ducks) compared with those who raised egg-laying or parent breeding stock, which can be attributed to several factors related to the industry’s value and associated biosecurity considerations [34].

Previous studies have identified a lack of “knowledge” as a crucial barrier to the adoption of recommendations for antimicrobial reduction in poultry production [26] or negligent biosecurity practices in cattle farming [14], as farmers are unaware of the effectiveness and, if any, economic benefits of doing so [12]. We found 33 producers for whom the Knowledge score was 3 or lower, indicating a lack of knowledge. This was particularly the case among producers of meat poultry.

Lack of knowledge and understanding, however, is not the only reason for a lack of biosecurity implementation [14,48,49]. Instead, it is often about recognizing the benefits associated with employing biosecurity measures [1]. This refers to Awareness and Desire. In our study, we found a low score (<=3) for Awareness in 10.3% and, for Desire, in 15.4% of the producers. In a study using a change model comparable to this one, authors found that a lack of awareness was preventing broiler producers from changing their antimicrobial usage for antimicrobial reduction [26].

The higher the education, the better the ADKA scores (Table 3), indicating a better understanding and willingness to implement farm biosecurity standards. Previous research in poultry [4], swine [50], and the dairy industry [51] supports this statement by showing that education influenced favorable attitudes and compliance with biosecurity policies.

Interestingly, it was also found that young producers had a greater understanding of the risks (Awareness) and had the possibilities (Ability) to achieve change, while middle-aged producers possessed more skills (Knowledge). On the contrary, the desire to change was unaffected by age difference but was influenced by the education level. Most (85%) poultry producers in the study expressed a desire to take action for improving biosecurity. Finally, gender did not have a significant impact on attitude scores.

Awareness of risks among farmers may not always lead to risk reduction behavior [52,53], mainly due to insufficient knowledge of measures against disease transmission, poor training, and lack of communication between workers and technical service providers [54]. According to a recent study [55], cattle farmers were less likely to change with regard to biosecurity due to satisfaction with their present situation and a tendency to underestimate the impact of the issue [56]. This illustrates that a positive mindset does not always transform into action, especially when farmers feel that their efforts will not make a difference [8]. Livestock farmers’ inclination and ability to invest in biosecurity measures might be influential factors [12,16]. This highlights the need to address both awareness and desire, because a deficiency in one or both of them may prevent any advice from being adopted.

The biggest challenge is to persuade farmers to change their practices to enhance biosecurity on their farms [4,50]. There is no “one-size-fits-all” approach to training personnel. Each worker possesses unique skill sets, experiences, and perceptions, and each farm has specific customs [16,57]. Therefore, efforts were made to enhance biosecurity compliance by using various intervention methods, such as coaching, group discussions, participatory meetings, etc., across the recruited farms during the course of the study. While participatory meetings, training sessions, and group discussions were considered beneficial for addressing general biosecurity concerns, coaching has been recommended for addressing more specific problems [27,28,58]. However, the challenge of coaching lies in the requirement for individuals who tend to change. In reality, not everyone is inclined to change. Participating in group discussions with like-minded individuals aiming for change can help overcome hesitations and be more effective in case where reluctance exists. In such cases, discussions at a group level are needed before transitioning to individual support. Furthermore, farmers are more likely to comply with recommendations when they have actively participated or invested their time in the process [59,60]. While coaching specifically relates to the farmer, group dynamics could vary, as certain farmers actively participate, while others passively observe during a group discussion. Nevertheless, every approach has specific benefits that may be more desirable depending on the farmer’s mindset and circumstances. Acknowledging the veterinarian’s role in farm biosecurity adds value [61].

A study conducted by de Carvalho Ferreira et al., 2024 [62] has demonstrated that the implementation costs for various types of supporting measures in participating farms were higher for farmers in participatory meetings, group discussions, and in-person training sessions. Conversely, individualized coaching at the farm required more time and higher cost for the coach. Further motivation may arise from this economic evaluation of comparable interventions.

The study results indicate that considering qualitative aspects is essential to structuring the approach, facilitating mutual understanding, and overcoming barriers to change. According to the authors, the choice between individual coaching and group approach can be made on the basis of the individual’s readiness for change. Nevertheless, after the implementation of the supporting measure, we observed a significant improvement in three of the four attitude elements. On average, the participating farmers experienced a positive change or overall benefit from engaging in this “teamwork” approach.

The goal is to disseminate information, acknowledging that farmers are free to choose whether or not to accept change. The farmers’ positive change, which was particularly noteworthy, was encouraging, especially considering that the guidance was coming from sources other than their usual source of information. The different kinds of interventions raised awareness about the importance of effective biosecurity and brought attention to potential vulnerabilities in biosecurity that the farmer was previously unaware of or did not regard as a possible danger. Furthermore, acknowledging job satisfaction and positive work–life balance like we did in this study can act as a strong motivator for implementing biosecurity practices [12,56] and improving animal health [17].

There are certain limitations to this study due to the study design that may impact the results. Firstly, poultry producer recruitment was based on convenience sampling. As poultry producers were selected based on their willingness to engage and give information, it is likely that they represented the better end of the population, possibly leading to a selection bias. Another shortcoming exists with regard to the comparison between production types and countries. As ADKAR scoring was performed by different researchers in different countries, we cannot guarantee perfect interobserver agreement. Moreover, it is possible that on some occasions, participants responded with socially desirable answers rather than their true beliefs [16], which may have had an overestimating effect on the outcome. In addition, producers’ experiences with the recent avian influenza outbreaks may have had a substantial impact on their perception of biosecurity at the time, explaining the scores obtained in this study.

## 5. Conclusions

This is a comprehensive study performed to understand the effect of socio-demographic factors on poultry biosecurity practices, demonstrating a significant link between specific demographic characteristics and attitude scores. To the best of the researchers’ knowledge, until now, there have been no studies in poultry producers across Europe investigating their attitudes, their understanding, and their motivations related to implementing biosecurity measures on their farms, using profiling tools. This study provides a foundation for future research into how veterinarians may help enhance biosecurity procedures on poultry farms. Individuals collaborating with poultry producers to promote biosecurity on poultry farms should be aware of the impact of the elements indicated in this study on biosecurity practice adoption. Following this, training should be structured to change the perception of producers about biosecurity practices.

## Figures and Tables

**Table 1:** Definition of scoring elements of ADKAR^®^ change management model.

ADKAR Building Block	Description Building Block (Element)	Score 1 = Lowest 5 = Highest	Explanation of Scores
**Awareness**	Represents the awareness that biosecurity in poultry production should be optimized to reduce the risk of the introduction and spread of infectious diseases.	1	Farmer misses all information regarding biosecurity and is not aware that improving biosecurity results in reduced risk of introduction and spread of infectious diseases/pathogens.
		2	Farmer is aware of the recommendation to improve biosecurity but is completely denying the potential effects of better biosecurity on risk of infectious disease introduction and spread.
		3	Farmer is aware that biosecurity should be improved but contests effects on animal health and production and mentions that disease introduction cannot be avoided anyway.
		4	Farmer is aware that biosecurity should be improved and positive effects on health and productivity of the flock are expected and accepts that some changes are required to achieve this.
		5	Farmer is fully aware that biosecurity should be improved, takes responsibility for biosecurity on the farm, and embraces the required improvement for the farm.
**Desire**	Represents the personification of awareness. “Does the farmer want to improve biosecurity on their farm?”	1	Farmer states the following: “This is not my problem. It does not concern me”.
		2	Farmer will improve but is not the first adaptor. Farmer states the following: “my neighbor should also improve”.
		3	Farmer wants to improve, but slowly. The goal is not to become the farm with the best possible biosecurity; just enough is also OK.
		4	Farmer’s goal is to improve biosecurity as much as possible, yet without substantial costs.
		5	Farmers goal is to improve biosecurity as much as possible, even if there are considerable costs related to this improvement.
**Knowledge**	Represents the knowledge and skills of the farmer to implement measures to improve biosecurity.	1	It is not clear what the risks of introduction and spread of infection on the farm are. It is not possible to draw up an action plan. The farmer and their network really do not know where to start.
		2	Either it is not known/understood which biosecurity improvements are required, or there is low or inaccurate knowledge, experience, or skills with regard to the execution of the biosecurity improvements.
		3	Information on the infection introduction risks is available and clear for the farmer, and an action plan can be drawn up.
		4	Information is available and clear, but some discussion about implementation is still present. Support for the farm and farmer is needed to implement change.
		5	Information is available and clear, the action plan is accepted, and knowledge and skills are sufficiently available at the level of the farmer and their network.
**Ability**	Represents the implementation phase of the change. Will or is the farmer implementing changes in biosecurity?	1	Farmers sees only obstacles for every proposed change and thus does not implement any.
		2	Farmer implements a limited number of changes which are easy to achieve. The selection is not made upon expected effect but on requested input.
		3	Some changes are accepted and implemented on the farm, or implementation is saved for rebuilding or a new building.
		4	Farmer is implementing systematically. But money or time is hampering some changes.
		5	Farmer is investing time, money, and/or effort to implement changes.
**Reinforcement**	Represents the sustainability of change. To sustain change, active positive reinforcement is necessary.	1	Farmer has negative experiences with improving biosecurity.
		2	Farmer received or receives negative feedback from the personal environment with regard to changed biosecurity measures.
		3	Improved biosecurity is not perceived to have a positive or negative effect.
		4	Improved biosecurity has led to more job satisfaction and better herd performance.
		5	Improved biosecurity has led to better economic performance or a higher personal status.

**Table 2:** Overview of supporting measures applied by each partner country.

Country	Supporting Measure
Belgium	On-farm individual coaching
Spain	On-farm individual coaching
Poland	Biosecurity training live workshop
France	Participatory group meetings or on-farm individual coaching
Italy	Virtual farm tour with group discussion and on-farm individual coaching
The Netherlands	Online sector meeting and online individual coaching
Hungary	Biosecurity training and on-farm coaching

**Table 3:** Descriptive information on the demographic variables age group, gender, and education, associated with the participants’ Awareness, Desire, Knowledge, and Ability scores.

Demographic Variables	N =	%	Awareness Score			Desire Score			Knowledge Score			Ability Score		
			$\bar{x}$ ± SD	≤3	≥4	$\bar{x}$ ± SD	≤3	≥4	$\bar{x}$ ± SD	≤3	≥4	$\bar{x}$ ± SD	≤3	≥4
				%	%		%	%		%	%		%	%
**Age group**														
<35 years old	36	23.8	4.4 ± 0.5 ^a^	2.8	97.2	4.3 ± 0.6	5.6	94.4	3.9 ± 0.8 ^a,b^	25	75	4.3 ± 0.6 ^a^	8.3	91.7
35–50 years old	72	47.7	4.3 ± 0.7 ^a,b^	12.5	87.5	4.1 ± 0.8	20.8	79.2	4.0 ± 0.8 ^a^	13.9	86.1	4.1 ± 0.8 ^a,b^	20.8	79.2
>50 years old	43	28.5	4.0 ± 0.6 ^b^	14.0	86.0	4.0 ± 0.7	16.3	83.7	3.7 ± 0.8 ^b^	32.6	67.4	3.8 ± 0.7 ^b^	32.6	67.4
**Gender**														
Male	106	77.9	4.2 ± 0.6 ^a^	8.5	91.5	4.1 ± 0.7 ^a^	16.0	84.0	3.9 ± 0.8 ^a^	21.7	78.3	4.0 ± 0.7 ^a^	20.8	79.2
Female	30	22.1	4.2 ± 0.7 ^a^	13.0	86.7	4.3 ± 0.7 ^a^	10.0	90.0	3.8 ± 0.8 ^a^	23.3	76.7	4.0 ± 0.7 ^a^	20.0	80.0
**Education**														
Lower	4	3.25	3.3 ± 0.5 ^a,b^	75.0	25.0	2.8 ± 1.0 ^a,b^	75.0	25.0	2.3 ± 0.5 ^a,b^	100.0	0	3.3 ± 0.5 ^a, b^	75.0	25.0
Higher	56	45.53	4.1 ± 0.5 ^b^	10.7	89.3	3.9 ± 0.7 ^b^	19.6	80.4	3.8 ± 0.6 ^b^	23.2	76.8	3.9 ± 0.7 ^b^	25.0	75.0
University	63	51.22	4.5 ± 0.6 ^c^	7.9	92.1	4.3 ± 0.7 ^c^	9.5	90.5	4.1 ± 0.8 ^c^	12.7	87.3	4.2 ± 0.7 ^c^	15.9	84.1

^a, b, c^ For each variable, values in the same column not sharing the same superscript are significantly different at *p* < 0.05 based on non-parametric Kruskal–Wallis test.

**Table 4:** Individual ADKAR^®^ profiles (for the elements Awareness, Desire, Knowledge, and Ability) of poultry producers (*n* = 155) rearing broilers, layers, turkeys, ducks, free-range broilers, and free-range layers; breeders; and hatcheries.

Demographic Variables	N =	%	Awareness Score			Desire Score			Knowledge Score			Ability Score		
			$\bar{x}$ ± SD	≤3	≥4	$\bar{x}$ ± SD	≤3	≥4	$\bar{x}$ ± SD	≤3	≥4	$\bar{x}$ ± SD	≤3	≥4
				%	%		%	%		%	%		%	%
Broiler producers	35	22.6	4.2 ± 0.6 ^a, b^	17.1	82.9	4.2 ± 0.7 ^a, b^	31.4	68.6	4.0 ± 0.7 ^a, b^	31.4	68.6	4.2 ± 0.8 ^a, b, c^	31.4	68.6
Layer producers	22	14.2	4.5 ± 0.6 ^a, b^	9.1	90.9	4.3 ± 0.6 ^a, b^	9.1	90.9	4.2 ± 0.6 ^a, b^	9.1	90.9	4.1 ± 0.7 ^a, b, c^	18.2	81.8
Breeders	24	15.5	4.6 ± 0.5 ^a^	4.2	95.8	4.4 ± 0.6 ^a, b^	12.5	87.5	4.2 ± 0.7 ^a, b^	8.3	91.7	4.5 ± 0.6 ^a, c^	8.3	91.7
Turkey producers	19	12.3	4.0 ± 0.6 ^b^	21.2	78.9	4.0 ± 0.8 ^a, b^	26.3	73.7	3.7 ± 0.9 ^a^	26.3	73.7	3.8 ± 0.7 ^b^	36.8	63.2
Duck producers	23	14.8	4.2 ± 0.5 ^a, b^	4.3	95.7	3.8 ± 0.6 ^a^	21.7	78.3	3.9 ± 0.6 ^a^	26.1	73.9	3.7 ± 0.6 ^a, b^	26.1	73.9
Free-range broiler producers	11	7.1	4.3 ± 0.5 ^a, b^	9.1	90.9	4.2 ± 0.7 ^a, b^	9.1	90.9	3.8 ± 1.1 ^a, b^	36.4	63.6	4.0 ± 0.7 ^a, b, c^	18.2	81.8
Free-range layer producers	11	7.1	4.3 ± 0.4 ^a, b^	0	100	4.2 ± 0.5 ^a, b^	18.2	81.8	4.0 ± 0.6 ^a, b^	27.3	72.7	4.3 ± 0.5 ^a, b, c^	0	100
Hatcheries	10	6.5	4.5 ± 0.7 ^a, b^	10	90	4.7 ± 0.5 ^b^	0	100	4.7 ± 0.5 ^b^	0	100	4.7 ± 0.5 ^c^	0	100

^a, b, c^ Values in the same column not sharing the same superscript are significantly different at *p* < 0.05 based on non-parametric Kruskal–Wallis test.

**Table 5:** ADKAR^®^ profiles of 130 poultry producers categorized based on the species of poultry they rear with mean scores (±standard deviation) for the elements Awareness, Desire, Knowledge, and Ability before and after implementation of supporting measures.

		Awareness		Desire		Knowledge		Ability		Reinforcement
Production Type	N =	Farm Visit		Farm Visit		Farm Visit		Farm Visit		Farm Visit
		1	3	1	3	1	3	1	3	3
		$\bar{x}$ ± SD	$\bar{x}$ ± SD	$\bar{x}$ ± SD	$\bar{x}$ ± SD	$\bar{x}$ ± SD	$\bar{x}$ ± SD	$\bar{x}$ ± SD	$\bar{x}$ ± SD	$\bar{x}$ ± SD
Enclosed broiler producers	31	4.1 ± 0.6	4.3 ± 0.5	4.2 ± 0.7	4.3 ± 0.7	3.9 ± 0.8	4.1 ± 0.6	4.1 ± 0.8	4.2 ± 0.8	3.7 ± 0.7
Enclosed layer producers	21	4.5 ± 0.7	4.7 ± 0.5	4.3 ± 0.6	4.4 ± 0.6	4.0 ± 0.7	4.3 ± 0.7	4.0 ± 0.8	4.1 ± 0.7	3.7 ± 0.7
Breeders	22	4.6 ± 0.6	4.6 ± 0.5	4.4 ± 0.7	4.5 ± 0.6	4.2 ±0.8	4.3 ± 0.8	4.4 ± 0.7	4.6 ± 0.6	4.1 ± 0.8
Turkey producers	18	3.9 ± 0.6	4.0 ± 0.6	3.9 ± 1.0	4.1 ± 0.8	3.6 ± 1.0	3.8 ± 0.9	3.8 ± 0.7	3.8 ± 0.7	3.4 ± 0.5
Duck producers	19	4.2 ± 0.4	4.3 ± 0.7	3.7 ± 0.8	3.9 ± 0.7	3.7 ± 0.7	3.9 ± 0.8	3.7 ± 0.7	3.6 ± 0.8	3.4 ± 0.6
Free-range broiler producers	10	4.2 ± 0.6	4.4 ± 0.7	4.2 ± 0.6	4.2 ± 0.9	3.9 ± 1.2	4.1 ± 0.9	3.9 ± 0.6	4.0 ± 0.9	3.7 ± 0.7
Free-range layer producers	9	4.1 ± 0.3	4.4 ± 0.5	4.0 ± 0.7	4.3 ± 0.5	3.7 ± 0.9	4.2 ± 0.7	4.2 ± 0.4	4.2 ± 0.4	3.4 ± 0.5
Total *	130	4.2 ± 0.5	4.4 ± 0.6	4.1 ± 0.7	4.2 ± 0.7	3.9 ± 0.9	4.1 ± 0.8	4.0 ± 0.7	4.1 ± 0.7	3.6 ± 0.6
*p*-Value		0.002 **		0.015 **		0.001 **		0.273		

* Comprises 130 participants who completed the study. ** The threshold for statistical significance was a *p*-value lower than 0.05.

## Data Availability

The data presented in this study are available upon request from the corresponding author. The data are not pub-licly available due to privacy and confidentiality agreements with the participants.
